# Improvement of DC Breakdown Strength of the Epoxy/POSS Nanocomposite by Tailoring Interfacial Electron Trap Characteristics

**DOI:** 10.3390/ma14051298

**Published:** 2021-03-08

**Authors:** Farooq Aslam, Zhen Li, Guanghao Qu, Yang Feng, Shijun Li, Shengtao Li, Hangyin Mao

**Affiliations:** 1 State Key Laboratory of Electrical Insulation and Power Equipment, Xi’an Jiaotong University, No. 28 Xianning West Road, Xi’an 710049, China; farooq@stu.xjtu.edu.cn (F.A.), lizhenl@stu.xjtu.edu.cn (Z.L.), guanghao_qu@stu.xjtu.edu.cn (G.Q.), iam1128@stu.xjtu.edu.cn (Y.F.), lishijunl@stu.xjtu.edu.cn (S.L.); 2 State Grid of Zhejiang Electric Power Co., Ltd., 347 Jiangjun Road, Hangzhou 310007, China; hangyinmao@126.com

**Keywords:** epoxy composite, POSS, DC breakdown voltage, charge carrier, charge traps, molecular simulation

## Abstract

To date, breakdown voltage is an underlying risk to the epoxy-based electrical high voltage (HV) equipment. To improve the breakdown strength of epoxy resin and to explore the formation of charge traps, in this study, two types of polyhedral oligomeric silsesquioxane (POSS) fillers are doped into epoxy resin. The breakdown voltage test is performed to investigate the breakdown strength of neat epoxy and epoxy/POSS composites. Electron traps that play an important role in breakdown strength are characterized by thermally stimulated depolarized current (TSDC) measurement. A quantum chemical calculation tool identifies the source of traps. It is found that adding octa-glycidyl POSS (OG-POSS) to epoxy enhances the breakdown strength than that of neat epoxy and epoxycyclohexyl POSS (ECH-POSS) incorporated epoxy. Moreover, side groups of OG-POSS possess higher electron affinity (*E*_A_) and large electronegativity that introduces deep-level traps into epoxy resin and restrain the electron transport. In this work, the origin of traps has been investigated by the simulation method. It is revealed that the functional properties of POSS side group can tailor an extensive network of deep traps in the interfacial region with epoxy and enhance the breakdown strength of the epoxy/POSS nanocomposite.

## 1. Introduction

With growing direct current high voltage (HVDC) applications, insulation failure has attracted much attention for epoxy-based insulating equipment to ensure power system reliability [1]. Hence, it is significant to explore the breakdown mechanism and improve the electrical performance of epoxy resin. In recent years, nanoparticle incorporation, which introduces a vast interfacial region between nanoparticles and polymer matrix, has attracted great attention in the insulation domain [2,3]. The interfacial region that occupies a large volume of nanocomposites can determine the dielectric strength [4]. Therefore, the breakdown performance of epoxy resin can be improved by nanoparticle incorporation, but the mechanism of breakdown relates to interfacial properties is still unclear.

In the interfacial region of nanocomposites, the dielectric strength is influenced by several factors, i.e., molecular chain dynamics, carrier traps, potential barrier of polymer structure against the charge transport, etc. [5,6,7]. In particular, carrier traps capture the charges that transport in the material are strongly related to the dielectric strength from recent studies [7,8]. Wang et al. studied the DC breakdown characteristics of LDPE/Al_2_O_3_ nanocomposites and found that the DC breakdown strength was dominated by deep trap level, which is improved by the incorporation of nanoparticles [9]. S. Li et al. reviewed numerous research on breakdown characteristics of nanocomposites, and finally established a positive correlation between dielectric strength and interfacial traps [10]. The traps can be tailored by incorporating conventional nanoparticles to enhance the breakdown strength of base polymers [11,12,13]. However, the existing incompatibility between inorganic nanoparticles and organic polymer, dispersion of particles, and uniform distribution is compromised, leading to the agglomeration of fillers. In order to avoid agglomeration and enhance compatibility, the particle’s surface is functionalized. Surfaces are treated with silane, grafting, and plasma treatment is utilized with varying degrees of achievement [14,15,16]. Alternatively, POSS could be a promising nanoparticle for electrical applications. It has a unique structure of inorganic Si–O central core with organic side groups attached to central core. These organic side groups enhance compatibility with multitude of polymers and form an interfacial region [17]. POSS can tailor interfacial traps by restructuration of the base polymer at the nanometric scale and has been recently reported for successful improvement of dielectric properties [7,18]. However, the origin of traps in the interfacial region and the effects of traps on breakdown performances need further investigations.

To investigate the charge trapping mechanism and explore the origin of traps that influences the DC breakdown strength of epoxy nanocomposites, two kinds of POSS fillers are incorporated into the epoxy matrix, and the DC breakdown strength of EP/POSS nanocomposites is studied. Trap characteristics are analyzed by thermally stimulated depolarization current curves, and molecular simulation techniques are used to calculate the electron affinity (*E*_A_), density of states (DOS), and frontier molecular orbital of EP/POSS nanocomposites, i.e., LUMO distribution. Finally, the origin of traps and the effects of traps on breakdown performance are discussed.

## 2. Materials and Experimental Methods

### 2.1. Materials

Epoxy resin Di-glycidyl ether of bisphenol-A type (DGEBA) was used as a matrix. Methyl tetrahydro phthalic anhydride (MeTHPA) was selected as a hardener. Epoxy/POSS composites were prepared by introducing two different types of POSS, i.e., octa-glycidyl POSS (OG-POSS) and epoxycyclohexyl POSS (ECH-POSS), respectively (Hybrid Plastics Inc. Hattiesburg, MS, USA). Both kinds of POSS features eight side groups attached to the silicon core, an accelerator (DMP-30) was used to fasten the curing process. The nomenclature of the samples is listed in Table 1.

### 2.2. Preparation of Epoxy/POSS Composites

**Figure 1:**
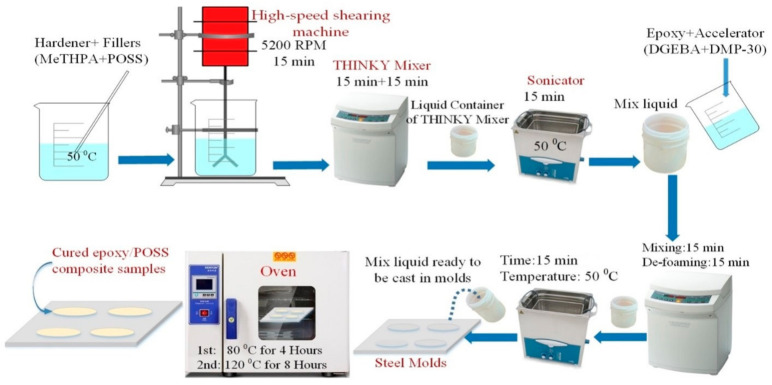
Fabrication process of epoxy/POSS nanocomposite samples.

**Figure 2:**
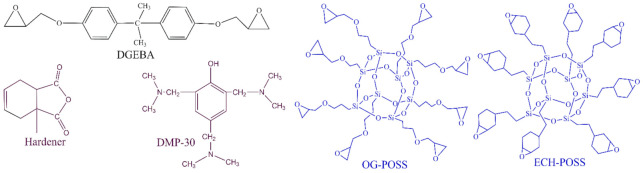
Molecular structure of epoxy (DGEBA), Hardener (MeTHPA), Accelerator (DMP-30), ECH-POSS and OG-POSS.

The fabrication process has been represented in Figure 1. First, DGEBA, POSS, and MeTHPA were kept at 60 °C for two days before blending to avoid bubbles and potential voids. POSS fillers were added into the curing agent MeTHPA (GH-9303, 1.21 g/cm^3^), and IKA T25 high-speed shearing machine was used to mix the liquid for 15 min with 5200 rpm speed. Then, the liquid is degassed and de-foamed by THINKY mixing machine for 30 min. To ensure the homogenous dispersion of POSS fillers into curing agent, sonication was performed for 15 min at 50 °C using an ultrasonic device of 99W power. Then, the DGEBA (*ρ* = 1.03 g/cm^3^) and DMP-30 accelerator was blended with the liquid. The same stirring and de-foaming procedures were repeated. Steel molds were cleaned by ethanol and kept in a kiln at 60 °C for 2 h before the liquid was cast into the different shapes and sizes of molds. An anti-setting agent was applied on the surface of molds to ensure an easy detachment of backed specimens. The mixed liquid was cured for 4 h at 80 °C and then for 8 h at 120 °C. The samples were then cooled to room temperature using an annealing process to get an adequately cured sample and to avoid cracks. The samples were cleaned with ethanol and then kept at 60 °C for 12 h before the experiments were carried out. A typical stoichiometric ratio for Epoxy, MeTHPA, POSS, and DMP30 was set as 100:80:2.5:1. POSS was added with a filler loading of 2.5 wt.% as an optimal value reported in Reference [19]. The chemical structure of DGEBA, MeTHPA, DMP-30, ECH-POSS, and OG-POSS is shown in Figure 2.

### 2.3. Breakdown Test

The DC Breakdown test was conducted using steel spherical electrodes in transformer oil at room temperature (25 °C). The diameter of spherical electrodes was 25 mm, and a computer-controlled DC voltage (HJC-100 kV) was applied with a ramp rate of 1kV/s. The sample with a thickness of 200 μm was selected for experiments. The DC breakdown data were statistically analyzed by two-parameter Weibull distribution. The cumulative probability of failure *P_i_* is expressed in Equation (1).

(1) $$P_{i}=1-\exp\left[-{\left(\frac{E_{bi}}{a}\right)}^{\beta }\right]$$

where *E_bi_* is experimental values of breakdown strength (kV/mm), *α* is a scale parameter that shows the cumulative failure probability of 63.2% samples in kV/mm, *β* is a shape parameter that reflects the variation of data. The *P_i_* of experimental data (*E_bi_*) can be approximated according to the IEEE-930 standard [20].

(2) $$P_{i}=\frac{i-0.44}{n+0.25}$$

**Figure 3:**
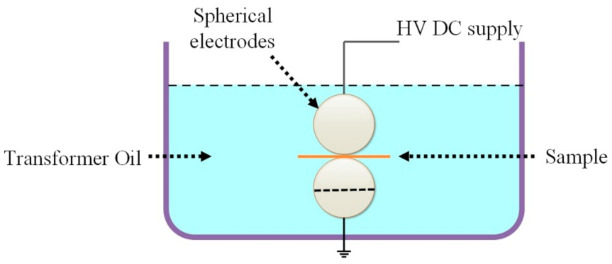
Schematics of breakdown voltage test.

Here, *i* represents the result of the *i*th rank, while ascending order is adopted for sorting all the values of *E_bi_*. *n* is total breakdown attempts, which are 16 in this work. The schematic is shown in Figure 3.

### 2.4. TSDC Experiment

The thermally stimulated depolarized current (TSDC) experiment was carried out to analyze trap characteristics of epoxy/POSS composites. Before the experiment, samples were gold-sputtered with a 30 mm diameter on both sides. Initially, the samples were polarized at 110 °C for 30 min under an applied electric field of 250 V. The sample was then abruptly cooled to 0 °C with −30 °C/min cooling rate and remained at this state for 10 min to ensure stable conditions. The applied electric field is removed, and the sample was short-circuited for a short time. The induced electrical charges remain inside the bulk. Finally, the sample temperature was increased with a ramp rate of 2 °C /min from 0 °C to 150 °C. After removing the applied electric field, the de-trapped carriers generate a depolarized current inside the bulk in the absence of an applied electric field. A Keithley 6517B (TEKTRONIX, INC. Shanghai, China) was used to measure the current *I(T)* as a function of linear temperature increase at rate *β*, expressed [21] as:

(3) $$I(T)=A\exp\left[-\frac{H}{kT}-\frac{\beta }{B}\int_{T_{O}}^{T}e^{-\frac{H}{k_{B}T}}dT\right]$$

Here, *T* is the absolute value in °C, *H* is trap energy of de-trapped electrons in eV, and *k_B_* is the Boltzmann constant (1.3802 × 10^−23^ J/K). Coefficient *A* and *B* are dependent on the model used for the TSDC curve, but independent of the *T* and *H*, which are in References [21,22].

(4) $$B=\frac{\beta H}{k_{B}T_{M}^{2}}\exp\left(-\frac{H}{k_{B}T_{M}}\right)$$

Here, *T_M_* is the corresponding temperature of peaks and *β* is the heating rate in °C/min. The number of trapped charges *Q_TSC_* at the depolarization stage can be calculated by the integration of the current peak.

(5) $$Q_{TSC}=\int_{T_{2}}^{T_{1}}I(T)dT$$

**Figure 4:**
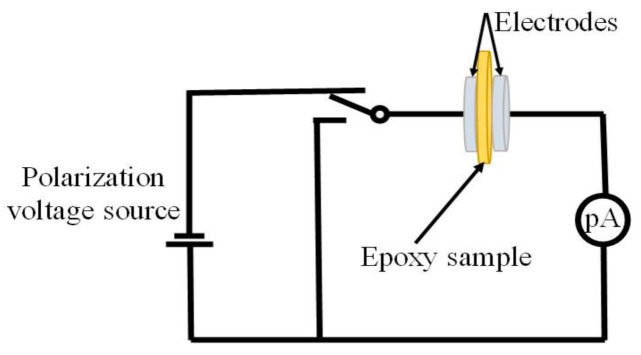
Schematics of thermally stimulated depolarized current (TSDC) test.

*T*_1_ and *T*_2_ are the initial and final temperatures of the peak, respectively. From the depolarized current, trap parameters *H* and *Q_TSC_* were analyzed. The schematic is shown in Figure 4.

### 2.5. Molecular Modeling and Simulation

#### 2.5.1. Schematics of Chemical Reaction

**Figure 5:**
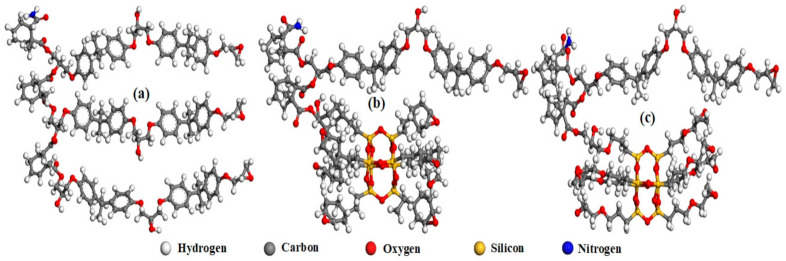
Molecular structure of (**a**) neat epoxy, (**b**) EP/ECH-POSS, and (**c**) EP/OG-POSS.

The DGEBA, MeTHPA, POSS, and DMP30 monomer molecules were constructed using Material studio software 2017 (San Diego, CA, USA) [23]. Based on the chemical reaction among DGEBA, hardener, and accelerator, the molecular structure of epoxy resin was built in Figure 5a. The presence of eight epoxycyclohexyl (ECH-POSS) and glycidyl (OG-POSS) side groups on the POSS core was the reason for selecting POSS as fillers for DGEBA, as shown in Figure 5b,c.

#### 2.5.2. Quantum Chemical Calculation (DFT Method)

To investigate the charge trapping sites of epoxy composites, ab-initio quantum chemical calculation using DFT method was employed using DMol3 package in Material Studio 2017. Based on the DFT calculation, the geometry optimization and energy calculations were investigated by the Perdew–Burke–Ernzerhof (PBE) function [24]. The computational work was accomplished with the basis set of double numerical plus polarization (DNP) and global orbital cutoff of 4 Å [25]. To incorporate the weak interaction between various fragments, the Grimme DFT-D dispersion correction method was employed [26].

## 3. Results

### 3.1. DC Breakdown Strength

**Figure 6:**
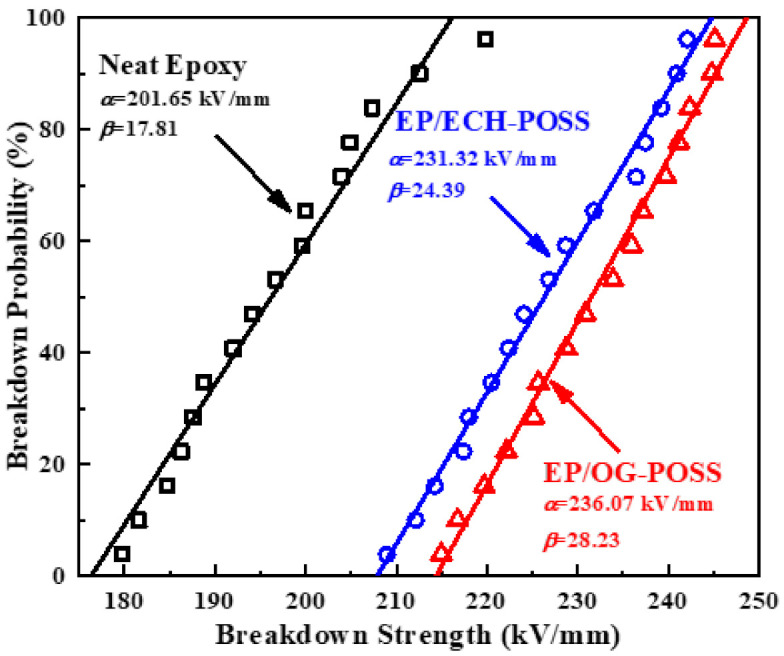
Weibull distribution plot for the breakdown test of neat epoxy, EP/ECH and EP/OG-POSS.

Weibull plots for the breakdown strength of epoxy/POSS nanocomposites are shown in Figure 6. Here, α is the scale parameter and shows the breakdown voltage strength in kV/mm for 62.3% samples that experience the electric breakdown. The shape parameter β represents an inverse measure of the breakdown data distribution. A higher β value means that the variation of breakdown data is lower for Weibull plotting. Both epoxy/POSS composites exhibit a significant increase in breakdown performance. The breakdown strength of neat epoxy is 201.65 kV/mm. While the breakdown voltages of EP/ECH-POSS and EP/OG-POSS are 231.32 kV/mm and 236.07 kV/mm, which are 15% and 17% higher than that of neat epoxy. Here a 2% slight retrogression is observed for EP/ECH-POSS, as compared to the EP/OG-POSS. The β values of EP/OG-POSS and EP/ECH-POSS are higher than that of neat epoxy samples, which illustrates that the breakdown data of epoxy/POSS has lower variations than neat epoxy. It is obvious that side groups of OG-POSS form perfectly tailored interfaces that offer hindrance to the mobility of charge carriers. These interfaces are home to deep traps. Deep traps have been investigated by the TSDC experiment. Incorporating POSS to epoxy can enhance the electrostatic potential and electron affinity for charge carriers, as compared to ECH-POSS. These factors increase the deeper traps of EP/OG-POSS and ultimately result in higher breakdown strength.

### 3.2. Trap Characterization by TSDC

**Figure 7:**
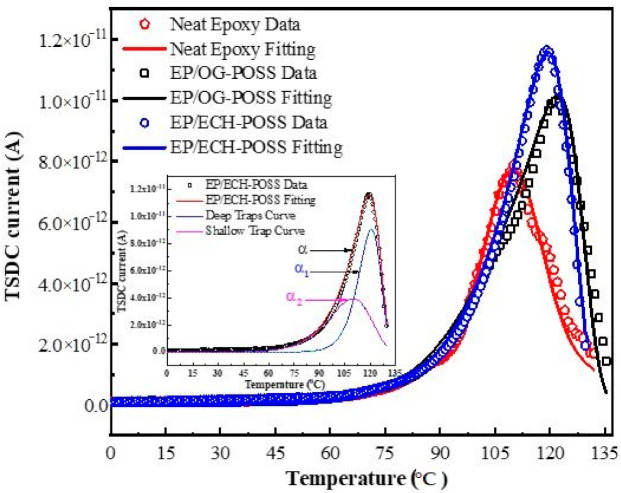
TSDC curves for all specimen (Dots for experimental results and lines for fitting curve). Inset: EP/ECH-POSS TSDC.

The TSDC results are shown in Figure 7. There are two obvious regions in the curve, i.e., the low-temperature region from 0 °C to 60 °C and the high-temperature region from 60 °C to 135 °C. An obvious peak is observed in the second stage with rising temperature. The peaks in the high-temperature region signify the de-trapping of charges and results in the TSDC current. This peak was characterized into deep and shallow traps peaks. For further interpretation of trap parameters, Equations (3) and (5) were utilized for TSDC curves fitting using MATLAB program. Table 2 shows the TSDC parameters for all three samples.

The fitting curves are consistent with experimental results in Figure 4. The *α* peak is characterized into two peaks, i.e., deep trap peak (*α*_1_) and shallow trap peak (*α*_2_), as shown in the inset figure of the EP/ECH-POSS sample. For epoxy/POSS composites, the trap levels are higher than that of neat epoxy. EP/OG-POSS has the highest deep trap energy of 2.15 eV. EP/ECH-POSS and neat epoxy have 2.0 eV, and 1.8 eV deep traps energy, respectively. It has been illustrated that tailoring deeper trap energy lowers the probability of trapped charges to escape. The deep trap has higher retention potency to restrain the trapped charges until some external factors are applied, such as temperature in this case is applied. Additionally, the TSDC peak for EP/OG-POSS occurs at higher temperatures. It shows that incorporating OG-POSS can form a large network of traps that restrain trapped electrons from de-trapping at earlier temperatures, as it requires external energy to escape. The neat epoxy has a smaller peak and a lower trap level (Table 2), indicating that fewer traps have been tailored; therefore, a lower peak has been observed. While the energy of shallow traps weakly links to the breakdown strength does not noticeably change for all samples (0.95 eV to 0.96 eV).

Table 2 shows the resultant trap parameters from the analysis of TSDC peak. The *Q_d_* and *Q_s_* show the quantity of de-trapped charges from deep and shallow traps calculated by Equation (5). The small peak of neat epoxy occurs at low temperatures, which indicates that trapped electrons escape at earlier temperatures. It means that only a low temperature is required to stimulate the trapped electrons to escape. In Table 2, values of *Q*_s_ and *Q*_d_ of neat epoxy are low, which causes the amount of de-trapped charges to be lower than the other two samples. For EP/ECH-POSS, it has the highest peak with large quantities of *Q_s_* and *Q_d_*, and its trap energy and corresponding temperature of peak are higher than neat epoxy. However, the trap’s energy of EP/OG-POSS is higher than EP/ECH-POSS, and the TSDC peak occurs at higher temperatures. In this case, it is difficult for electrons to escape from deep traps in EP/OG-POSS.

### 3.3. Density of States (DOS) and Energy Level Distribution

**Figure 8:**
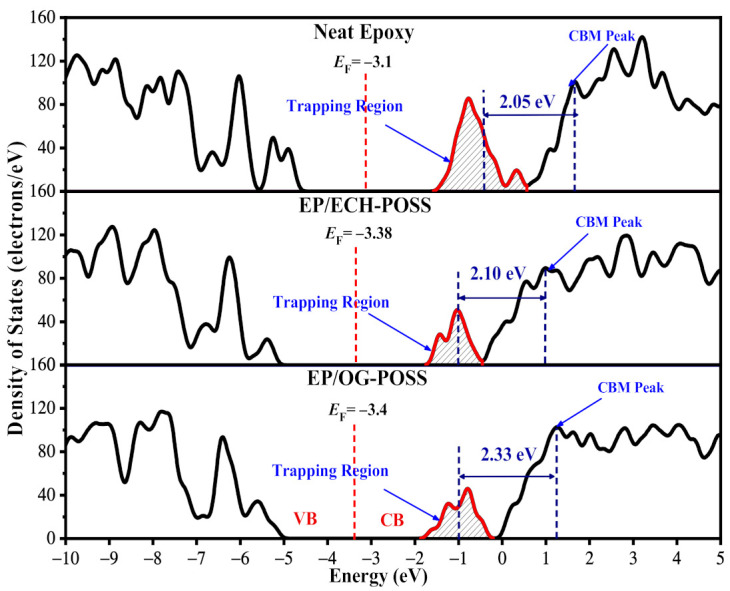
Total density of states (TDOS) of neat epoxy, EP/ECH-POSS, and EP/OG-POSS. Black line represents TDOS. *E*_F_ is shown by vertical red dashed line in the mid-point of valance band (VB) and conduction band (CB). The trapping region is shaded. The first peak of CB is regarded as the CBM peak.

Figure 8 shows the total density of states (TDOS) of EP/ POSS nanocomposites. DOS is the available states for an electron to occupy at a certain energy level. There is a broad gap in the Fermi energy level (EF) vicinity, where the DOS is zero. The boundaries of the gap are the valance band (VB) and conduction band (CB), while the *E*_F_ is located in the middle of the bandgap. There are many peaks in the whole energy level. On the right side of *E*_F_, the first peak (shaded) is recognized as the trapping peak, while the second peak is the bottom of the CB. Hence, a number of traps accumulate near the CB, and the gap distance between the center of the trapping peak and the CBM peak is defined as trap depth. For neat pristine epoxy resin, the trap depth is 2.05 eV. The trap depth is improved by the introduction of POSS to the epoxy matrix. For EP/ECH-POSS, the trap depth increases by 0.05 eV compared to neat epoxy resin, while it increases 0.28 eV for EP/OG-POSS. It is obvious that EP/OG-POSS has a larger trap depth.

Shaded areas in Figure 8 under the red curve near *E*_F_ are regarded as the trap density. Referring to Figure 5, the neat structure has three DGEBA chains, while the other two samples have only one DGEBA chain. The trapping region of the neat sample is three times larger than the other two samples. To compare the shaded area for all samples, the area under the curve for neat is multiplied with a factor of 0.3. The outcome shows that the trapping region for EP/OG-POSS is 33.02 A^2^, which is larger than the EP/ECH-POSS and pristine sample, i.e., 31.8 A^2^ and 24.1 A^2^, respectively. The trap characteristics of EP/POSS nanocomposites calculated by TDOS are consistent with TSDC results.

**Figure 9:**
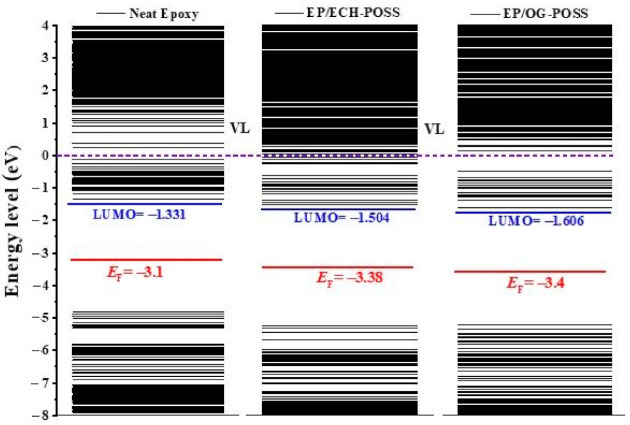
Energy level distribution of samples. Redline is *E*_F_, blueline is LUMO, and violet line is VL.

Figure 9 represents the energy level distribution of EP/ POSS nanocomposites. The lowest unoccupied molecular orbital (LUMO) and highest occupied molecular orbital (HOMO) were derived from DOS calculation. The mid-position of HOMO and LUMO is *E*_F_. The vacuum level (VL) is at the zero energy level, and the gap distance between VL and LUMO is the electron affinity (*E*_A_), which is the released energy when the composite acquires an electron and closely associated with the trapping depth in the bulk of the matrix [27]. For neat epoxy resin, the *E*_A_ is 1.331 eV. While for EP/ECH-POSS and EP/OG-POSS, the *E*_A_ is 1.504 eV and 1.606 eV, which is 0.173 eV and 0.275 eV higher than neat epoxy.

## 4. Discussion

### 4.1. Relationship between DC Breakdown and Traps

**Figure 10:**
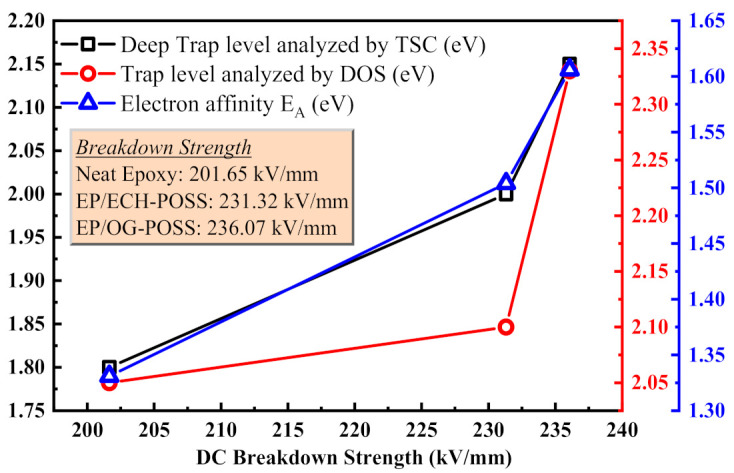
Relationship between DC breakdown strength and trap characteristics analyzed by TSDC test and molecular simulation.

Figure 10 shows the relationship between DC breakdown strength and trap characteristics analyzed by TSDC and molecular simulation. The trap parameters have been taken from TSDC analysis, DOS calculations, and energy distribution diagram. As the deep trap level and electron affinity (*E*_A_) increase, the DC breakdown strength increases, which indicates that the trapping mechanism strongly influences the breakdown strength. DC breakdown strength has a positive relationship with the trap level, and *E*_A_ is calculated from the energy distribution diagram in Figure 9. The results show that doping of the OG-POSS filler improves the trap level and *E*_A_, which eventually enhances the DC breakdown strength.

### 4.2. Origin of Traps Introduced by POSS Nanofillers

**Figure 11:**
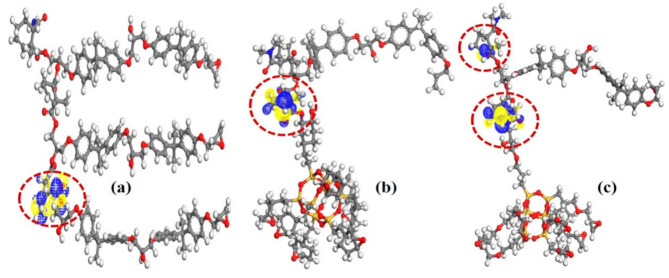
LUMO distribution of (**a**) Neat epoxy, (**b**) EP/ECH-POSS, and (**c**) EP/OG-POSS.

Figure 11 shows the LUMO distribution of pristine EP/ECH-POSS and EP/OG-POSS. In these three molecular structures, the isosurface of LUMO exists in the interaction region’s vicinity between MeTHPA and epoxide groups. It is shown that the hardener and its bonded chemical groups contributed to the LUMO level of epoxy and its composites. Based on nanocomposites multi-core model, the bonded region is the innermost interfacial region [4]. The LUMO level promptly captures electrons, which acts as a trap center and plays a vital role in manipulating electron transport. Likewise, it is determined that deep-level traps of nanocomposite originate in the interfacial region of EP and POSS.

A molecular structure containing a large number of electronegative atoms has higher electrostatic potential (ESP). Higher ESP positively influences the dominant features of DOS and energy level distribution [18]. Chemical groups attached to POSS central core play a decisive role in tailoring traps in bulk. Moreover, additional crosslinking sites are introduced by doping chemical groups with higher functional properties, eventually form a potential trapping center in the polymeric bulk [7].

### 4.3. The Effect of Traps on the DC Breakdown Performance

Traps play an important role in DC breakdown performance as confirmed from Section 4.1. In general, traps mainly dominate three physical processes in dielectric breakdown: charge injection, charge migration, and breakdown criterion. At a high electric field, the injection of electrons near the cathode is determined by the work function *Φ*_0_ of electrodes and the applied field *E*, and the potential barrier *Φ*_1_ for electrons injection can be calculated by the Schottky effect:

(6) $$\phi_{1}=\phi_{0}-\beta_{1}\sqrt{E}$$

Here, *β*_1_ is a coefficient of Schottky effect, which can be expressed as:

(7) $$\beta_{1}=\sqrt{e^{3}/4\pi \varepsilon_{0}\varepsilon_{r}}$$

Here *e* is the charge quantities of an electron (1.602 × 10^−19^ C), *ε*_0_ is permittivity in vacuum (8.854 × 10^−12^ F/m) and *ε*_r_ is relative permittivity.

**Figure 12:**
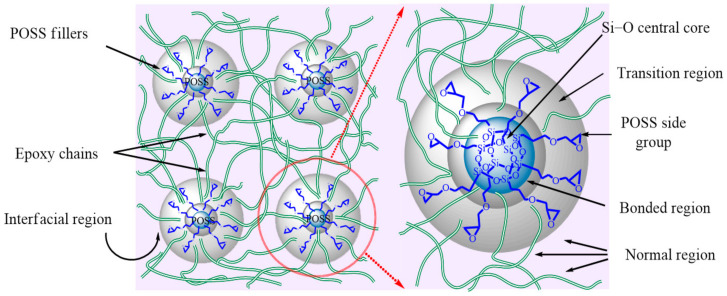
Modified morphology epoxy/POSS composite in the interfacial regions and POSS distribution.

Figure 12 shows the morphology and interfacial region of POSS and epoxy matrix. For EP/POSS nanocomposites, large amounts of traps are introduced. As the applied field increases with time, some “hot-electrons” jump over the barrier and are captured by the traps, and lots of homo-space charges are accumulated near the electrodes, which impedes further charge injection process. In this case, the electric field distortion is not served through the material, and the DC breakdown is hard to be triggered. Both the trap level and the *Q_TSC_* improved by POSS reduce the space charge and enhance the DC breakdown strength, which is consistent with the phenomenon in Figure 10.

The charge migration process under high-applied field is also associated with the traps in the EP/POSS nanocomposites. For the charges, the trapping and detrapping probabilities take the form of:

(8) $$P_{tr}=\frac{eN_{t}\mu_{0}}{4\varepsilon_{0}\varepsilon_{r}}$$

(9) $$P_{\mathrm{de}}=\nu_{ATE}\exp\left(-E_{t}/k_{B}T\right)$$

where *P_tr_* and *P_de_* are the trapping and detrapping probabilities, *E_t_* and *N_t_* are the trap level in eV and trap density in m^−3^, *μ*_0_ is carrier mobility in m^2^·V^−1^·s^−1^, and *υ_ATE_* is the attempted escape frequency in s^−1^. In Equations (8) and (9), the *P*_de_ decreases with the trap level, which indicates that the trapped charges in deep trap cannot gain enough energy to detrap unless the applied field is improved, while the increase of trap density improve the *P*_tr_ and capture more charges into the trap. Hence, the increases of trap level and density suppress the charge transport process in the dielectric material, reducing the conductivity and improving the dc breakdown strength. In addition, the charge transport process in dielectric material is determined by Poole-Frenkel effect [28]. The potential barrier *Φ*_1_ for charge function is the same as Equation (6), but the *Φ*_0_ is the trap level and *β*_1_ is the coefficient for the Poole-Frenkel effect:

(10) $$\beta_{1}=\sqrt{e^{3}/\pi \varepsilon_{0}\varepsilon_{r}}$$

In this case, the increase in trap level by POSS suppresses the charge transport process in dielectric and further improves the dc breakdown strength for EP/POSS nanocomposites.

**Figure 13:**
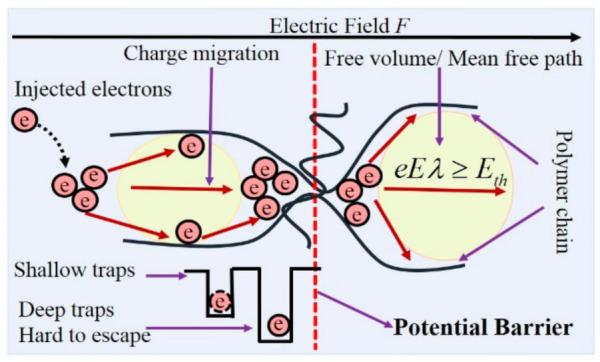
Schematic of breakdown model based on the potential barrier.

Figure 13 shows the potential barrier against the charge transport tailored in the polymer matrix. For epoxy resin, there are large areas of amorphous regions and molecular chains are easily moved in the amorphous region. In this case, free volume breakdown is a potential dielectric failure mechanism for epoxy composites at room temperature [29]. In the free volume approach, breakdown is triggered as the electrons are accelerated by applied field and hop over the potential barrier. In this case, the breakdown strength *E* is dominated by the longest free path *l*_st_, and the height of potential barrier *E*_th_, the free volume breakdown criterion can be expressed as:

(11) $$Eel_{st}\geq E_{th}$$

Here *E_th_* is the trap level in this work, which indicates that the breakdown strength is improved by the increase in deep trap level.

Furthermore, the dc breakdown characteristics in the epoxy nanocomposites are not only dominated by the trap characteristics. The free volume length, the potential barrier around the nanoparticles, and the scattering effects of nanoparticles are all potentially influencing factors for dielectric failure. All the changes in trap characteristics, local electrical field, and space charges may affect the dc breakdown characteristics for epoxy composites. From this work, a reliable path is provided to improve the DC breakdown strength by tailoring the traps through nano-structured POSS incorporation.

## 5. Conclusions

In this study, two different types of POSS were incorporated into the epoxy matrix. The influence of POSS side groups on tailoring the charge traps and their impact on breakdown strength was investigated. The trapping phenomenon in polymeric insulators was studied by the experimental results of TSDC and simulation results by TDOS and energy distribution diagram. Based on the experimental and simulation results, the main conclusions are as follows:

1. Incorporation of OG-POSS and ECH-POSS nanofillers to epoxy matrix successfully increases the deep trap level and breakdown strength, the DC breakdown strength of EP/OG-POSS, and EP/ECH-POSS increases 17% and 15% compared to neat epoxy.
2. A positive relationship has been established between DC breakdown strength and trap parameters, i.e., deep trap level and electron affinity (*E*_A_).
3. Traps originated from the interfacial bonded region of EP/POSS. The depth of the trap level has a positive correlation with the electronegativity of atoms in the side groups of POSS. The greater the electronegativity of polymeric composite, the larger would be the trap depth.
4. The increment in deep trap level and density suppresses the charge injection and transport process in the dielectric material, restraining the carriers from hopping over the potential barrier and further improves the DC breakdown strength.

## Figures and Tables

**Table 1:** Ingredients and sample code.

Sample Code	Sample Ingredients
Neat epoxy	Epoxy (DGEBA + MeTHPA + DMP-30)
EP/OG-POSS	Epoxy+OG-POSS 2.5%
EP/ECH-POSS	Epoxy+ECH-POSS 2.5%

**Table 2:** Trap parameters of EP/POSS composite using TSDC.

	α Peak1 (α1)			α Peak2 (α2)		
Samples	Trap Energy/eV	*Q_d_* (nC)	T_m1_ (°C)	Trap Energy/eV	*Q_s_* (nC)	T_m2_ (°C)
Neat	1.8	1.12 × 10^−9^	108	0.95	1.58 × 10^−9^	96
EP/ECH-POSS	2	8.11 × 10^−9^	124	0.96	6.74 × 10^−9^	116
EP/OG-POSS	2.15	6.69 × 10^−9^	127	0.956	6.83 × 10^−9^	116

## Data Availability

Data sharing is not applicable to this article.
